# *Schistosoma bovis* Infecting Humans in Nigeria

**DOI:** 10.4269/ajtmh.24-0714

**Published:** 2025-01-07

**Authors:** Oluwaremilekun G. Ajakaye, Elisha E. Enabulele

**Affiliations:** Department of Animal and Environmental Biology Adekunle Ajasin University Akungba Akoko Ondo State, Nigeria; Laboratory of Molecular Parasitology and Genomics of Neglected Tropical Diseases Adekunle Ajasin University Akungba-Akoko Ondo State, Nigeria E-mail: remilekunf@gmail.com; Laboratory of Integrative Parasitology and Pathogen Genomics Texas, USA E-mail: enabuleleegie@gmail.com

Dear Editor,

We read the article entitled “*Human Schistosomiasis due to Schistosoma bovis in Nigeria*” by Enudi et al., published in *The American Journal of Tropical Medicine and Hygiene*.^1^

In the article, an *S. bovis* cytochrome c oxidase subunit 1 (COX1) gene profile was identified in a pool of eggs from a human sample. It was suggested that *S. bovis* could infect human beings. Unfortunately, the authors did not include a nuclear marker in their genetic analysis. The authors also used a pool of *S. haematobium* eggs for DNA extraction. Ideally, a single egg is more appropriate to avoid genotyping a mixed *Schistosoma* species infection.

The inference that *S. bovis* was the causative agent of human schistosomiasis was based on an incomplete dataset. *Schistosoma bovis* COX1 profiles have previously been identified in samples from Nigeria^2^^,^^3^ and other parts of Africa alongside an *S. haematobium* internal transcribed spacer (ITS) profile representing mitonuclear discordance, resulting from either ancient or extant hybridization between *S. bovis* and *S. haematobium*.^4^ It is also important to highlight that two mitotypes, *S. bovis* and *S. haematobium,* can sometimes be recorded in *S. haematobium* populations, particularly in West Africa.^2^^,^^5^^,^^6^

Although the COX1 and ITS markers are traditionally used to genotype *Schistosoma* species, there are suggestions that the markers are inadequate, and scientists genotyping based on the two markers alone should interpret their results with caution.^2^^,^^6^ Therefore, the title and conclusion of the article are potentially misleading without the nuclear marker profile for the sample reported.
